# Pre and postnatal exposure to Chikungunya virus does not affect child neurodevelopmental outcomes at two years of age

**DOI:** 10.1371/journal.pntd.0008546

**Published:** 2020-10-05

**Authors:** Randall Waechter, Erinique Ingraham, Roberta Evans, Nikita Cudjoe, Amy Krystosik, Rashida Isaac, Ashlee Watts, Trevor Noël, Barbara Landon, Michelle Fernandes, Veronica Mapp-Alexander, Priyanka Suresh, George Mitchell, Calum Macpherson, Patrick Gérardin, A. Desiree LaBeaud

**Affiliations:** 1 Department of Neuroscience and Physiology and Behavioral Sciences, School of Medicine, St. George’s University, St. George’s, Grenada, West Indies; 2 Windward Islands Research and Education Foundation, St. George’s, Grenada, West Indies; 3 Stanford University, School of Medicine, Department of Pediatrics, Division of Infectious Disease, California, United States of America; 4 Office of Research, St. George’s University, St. George’s, Grenada, West Indies; 5 Psychological Services Center, St. George’s University, St. George’s, Grenada, West Indies; 6 Faculty of Medicine, Department of Paediatrics, University Hospitals Southampton, University of Southampton, Southampton, United Kingdom; 7 Nuffield Department of Women’s & Reproductive Health, John Radcliffe Hospital, University of Oxford, Oxford, United Kingdom; 8 School of Veterinary Medicine, St. George’s University, St. George’s, Grenada, West Indies; 9 Office of Chief Medical Officer, Ministry of Health, St, George’s, Grenada, West Indies; 10 INSERM CIC1410, Centre Hospitalier Universitaire de la Réunion, Saint Pierre, Réunion / Unité Mixte 134 PIMIT (Université de La Réunion, CNRS 9192, INSERM U1187, IRD 249), Sainte Clotilde, Réunion; University of Costa Rica, COSTA RICA

## Abstract

**Background:**

The 2005–06 chikungunya virus (CHIKV) outbreak in La Réunion suggested that mothers could transmit CHIKV to their neonates while viremic during the intrapartum period, and more than half of the infected neonates showed impaired neurodevelopment at two years of age. However, data sparsity precluded an overview of the developmental impact of vertical infection within the whole prenatal period.

**Objective & methods:**

The current study assessed two-year old children born to mothers who were infected during the 2014 CHIKV outbreak in Grenada to determine the neurodevelopmental impact of perinatal CHIKV infection throughout gestation. Mother and child infection status were confirmed by serologic testing (IgG and IgM) for CHIKV. Cognitive, fine motor, gross motor, language and behavioral outcomes were assessed at two years of age on the INTERGROWTH-21^st^ Neurodevelopment Assessment (INTER-NDA).

**Results:**

No differences in neurodevelopmental outcomes were observed between two-year-old children born to mothers infected with CHIKV during gestation (n = 149) and those born to mothers not infected with CHIKV (n = 161). No differences were found in INTER-NDA scores between children infected with CHIKV (n = 47) and children not infected with CHIKV (n = 592). Likewise, there were no differences between children infected with CHIKV post-partum (n = 19) versus children not infected with CHIKV (n = 592).

**Conclusion:**

Our findings suggest that children exposed and/or infected with CHIKV outside of the intrapartum period experience no significant neurodevelopmental delay at two years of age, as measured by the INTER-NDA, compared to their unexposed and/or uninfected peers. These results complement those of previous studies which showed a neurodevelopmental risk only for children infected during the intrapartum period, while the mother was highly viremic. These results might be reassuring for women of childbearing age and public health officials in CHIKV-endemic regions.

## Author summary

The Chikungunya virus (CHIKV) can negatively impact fetal neurodevelopment and consequent neurocognitive functioning in young children. Studies from La Réunion island demonstrated that, compared to their uninfected counterparts, children infected by CHIKV during the intrapartum period were at increased risk for deficiencies in the frontal lobes where the language and coordination centers are located, as well as slight deficits in other neurocognitive skills like sociability, movement and posture. The current study assessed two-year old children born to mothers infected during the 2014 CHIKV outbreak in Grenada to determine the neurodevelopmental impact of perinatal infection throughout gestation. No differences in neurodevelopmental outcomes were observed between two-year-old children born to mothers infected with CHIKV during gestation and those born to mothers not infected with CHIKV, nor between children infected with CHIKV and children not infected with CHIKV. Our results support previous findings that a small proportion of children born in CHIKV-endemic regions are at risk as only mothers viremic during the intrapartum period are at risk of infecting their neonates. Healthcare providers in CHIKV-endemic regions may wish to monitor for CHIKV symptoms when pregnant women are near term. The relatively high symptomatic rate seen with CHIKV infection (75%) makes such monitoring feasible.

## Introduction

The chikungunya virus (CHIKV) was first documented in southern Tanzania in 1952 [1]. Reports of the virus were contained to the African continent until it spread to Asia between the 1960’s– 1980’s [2]. In 2004, CHIKV spread to previously non-endemic areas including Southern Europe and Latin America [2–4]. At the end of 2013, CHIKV was first reported in the Caribbean region, with over 4,300 confirmed cases in St. Maarten, French Guiana and neighboring Caribbean countries [2,5], including Grenada [6]. The spread of CHIKV was driven by a number of factors including immunologically naïve populations, climate change, globalization, urbanization and increasing trade and travel between tropical and subtropical regions [3,4,7]. CHIKV has posed a significant threat to regional public health because of its rapid spread, limited diagnostic capabilities, continued unreported/undetected circulation throughout Latin America and the Caribbean [4,5] and long-term health consequences [8,9].

Transmitted to humans via the bite of an *Aedes aegypti* or *Ae*. *albopictus* mosquito vector [4], CHIKV replicates quickly in the human body, targeting connective tissue, epithelial and endothelial cells, fibroblasts, macrophages and muscle progenitor cells causing a range of clinical manifestations: high fever, arthralgia, myalgia, rash, headache, nausea, and vomiting [10–13]. In contrast to many other arboviral infections, only 5–25% of CHIKV infections are asymptomatic, facilitating syndromic surveillance during a CHIKV outbreak [3]. Distinct symptoms have been reported in females (joint pain, rash and joint swelling) compared to males (chills and fever) [6]. Severe neurologic manifestations, such as encephalitis and Guillain Barré syndrome, have also been reported [3,10,14–17]. While most cases of CHIKV are non-fatal, there have been deaths [3,5] and excess mortality [18] associated with the infection in almost all areas where the virus has spread including the Indian ocean, the Caribbean and Latin America.

Viremic mothers can transmit CHIKV infection to their neonates during childbirth [19]. In June 2005, the first mother-to-child CHIKV transmission was identified in La Réunion. By April 16, 2006, 38 cases of vertical transmission had been reported in infants less than 10 days of age [20]. Since neonates and infants are more susceptible to adverse clinical manifestations by the virus, they tend to have a different clinical presentation compared to older children and adults [15,21]. Neonates typically become symptomatic at four days old with symptoms of sepsis including fever, pain, hypotonia, edema, respiratory difficulties like apnea, thrombocytopenia, and symptoms of and MRI findings consistent with encephalitis and disseminated intravascular coagulation (DIC) [15,17,19,22,23]. Infants may also experience febrile seizures, diarrhea, vomiting, arthralgia/arthritis, acrocyanosis, severe bullous, maculopapular and erythematous macular skin lesions, hypo- or hyperpigmentation, uremia, lymphopenia, cytolysis, cardiovascular decompensation, secondary bacterial infections and vital organ damage and failure including necrotizing enterocolitis, myocarditis, and pericarditis [10,13,15–17, 19,21,24,25].

The limited availability of data on CHIKV in neonates makes it challenging to describe the rates and mode of mother-to-child transmission and subsequent developmental impact of the virus. Some clinical syndromes such as abnormal cerebrospinal fluid (compatible to encephalitis, meningitis, or meningoencephalitis), encephalopathy, neuronal loss, microcephaly and central nervous system diseases like cerebral palsy, seizures and Guillain-Barré syndrome have been linked to poor neurodevelopment in infected neonates [10,13–16,19,23,26]. Follow up studies from the La Réunion CHIKV outbreak demonstrated notable neurocognitive impairment for children who were perinatally infected compared to their uninfected counterparts, including deficiencies in the frontal lobes where the language and coordination centers are located and slight deficits in other neurocognitive skills like sociability, movement and posture [14]. Additionally, severe cases may present a progressive decrease of cerebral and cerebellar hemorrhages and replacement of brain edema features by subsequent demyelination of the white matter [15]. Despite the severity of symptoms and their neurological impact, neonates who did not experience neurological complications recovered in two to three weeks without sequelae [16].

The current study complements published data on mother-to-child transmission and the subsequent impact of CHIKV on child neurodevelopmental outcomes by assessing two-year old children born to two groups of pregnant mothers: those who were infected and those not infected with CHIKV during the outbreak in Grenada in 2014. Neurodevelopment was assessed on the INTERGROWTH-21^st^ Neurodevelopment Assessment (INTER-NDA) which measures cognitive, fine motor, gross motor, language and behavioral domains [27]. The INTER-NDA was chosen for test item accessibility in a low-middle income country (LMIC), its non-specialist administration and brief assessment time (i.e., 15–20 minutes).

## Methods

### Ethics statement

This study was approved by the Institutional Review Board of St. George’s University (#16026), Stanford University (#37739) and through agreement with the Grenada Ministry of Health. Written informed consent was obtained from all study participants.

### Study site and location

The CHIKV outbreak in Grenada, West Indies occurred between July and November 2014 (6). In partnership with the Grenada Ministry of Health, an enumeration list of all births in Grenada between 2014–2016 was created from birth records and the telephone contact of the mother extracted. Mother-child dyads were eligible for inclusion if the woman gave birth between January 1, 2014—February 28, 2016 (6 months prior to the outbreak to 15 months post outbreak) and the child was living with the mother at the time of the study enrollment.

### Procedures

As children reached 22 months of age, a member of the research team telephoned all eligible mothers to explain the study and invite her to meet with a trained and qualified research assistant who explained the study and obtained written informed consent. Following consent, the mother completed surveys detailing the onset and symptoms related to her CHIKV infection on an electronic tablet. Due to varying levels of participant literacy and the sensitive nature of some of the items, the questionnaires were sometimes administered to the mothers by the research assistants, who recorded responses. A second research assistant administered a comprehensive developmental evaluation, the INTER-NDA, to the child. Whole blood was obtained from both mothers and children via venipuncture by registered nurses. Blood was transported in ice-packed coolers and refrigerated at 4°C and serum separated within 24 hours and frozen at -80°C.

### Materials

Questionnaire assessment: The following outcomes assessments were administered during the single study visit:

1. Clinical questionnaire detailing risk factors, signs, and symptoms of CHIKV infection. This questionnaire included items that asked about the participants’ contact with mosquitoes and their medical history and symptoms of arboviral diseases (e.g., chikungunya, dengue, yellow fever). Demographic information and mosquito-borne illness symptoms during pregnancy were also collected in this measure.
2. Infant birthing questionnaire (IBQ)–This survey assessed the health and experiences of the mother during pregnancy, complications during the birthing process, and any health problems in the newborn babies for the first 2–3 weeks following birth. It also examined any potential postnatal risk factors by inquiring about the mother’s drug and alcohol use during pregnancy.
3. The General Health Questionnaire (GHQ-12)–A screening instrument that is used to detect current psychological distress/mental health, with the exception of psychotic psychiatric disorders [28]. Twelve items screen for three overarching problems: depressed mood, anxiety, and social dysfunction. Respondents rated how much they have recently experienced each of the problems on a 4-point scale from “much less than usual” to “more than usual” that focused on two major areas: 1) the inability to carry out normal functions, and 2) the appearance of new and distressing phenomena. The GHQ-12 is a quick, reliable and sensitive short form, making it ideal for research studies [28].
4. The Home Observation for Measurement of the Environment (HOME) questionnaire–The HOME is a well-established measure of parental support and the child’s experiences in his/her home environment [29]. Data is collected via a series of observation and direct interview items. The 45-item questionnaire is divided into six subscales: (i) parental responsivity: the degree of responsiveness of the parent to the child; (ii) acceptance of the child: parental acceptance of less than ideal behavior and avoidance of restriction and punishment; (iii) organization of the home environment: predictability of the home environment (including regular routines); (iv) learning materials: provision and availability of suitable learning items (v) parental involvement: the degree to which parents are involved with their child; and (vi) variety in the home atmosphere. These subscales provided a snapshot of the immediate environment of the children including their experiences, parental support and available resources [29].
5. The USDA Household Food Insecurity Access Scale (HFIAS) for Measurement of Food Access–The third version of the food security scale is designed to assess the prevalence and degree of food security in participants’ households. It consists of 18 questions that examine availability, description of and consumption of food in participants’ homes. The questions also measure feelings, behaviors and consequences associated with food security [30,31].
6. The Social Support Questionnaire (SSQ)—The SSQ, adapted from the Assessment of Parental Wellbeing and Behaviours [32], is a 12 self-report item survey aimed at assessing perceived levels of social support. It consists of six informational items that assess regular access to instrumental support and six items on emotional support and positive appraisal. The adaptation of the measure used in the current study has an internal consistency of 0.88 [32].
7. The INTERGROWTH-21^st^ Neurodevelopment Assessment (INTER-NDA) (27,33) is a multi-dimensional, standardized assessment measuring cognition, motor, language and behaviour outcomes in children aged 22 to 30 months. It was developed for and has been implemented in low-, middle- and high-income populations [27]. Its 37 items are scored on a 5-point scale, characterizing the child’s performance across a spectrum rather than on the traditional binary (pass/fail) outcome employed by some contemporary developmental tests (e.g., the Bayley Scales of Infant Development, BSID) [34]. It utilizes a mixed methodology psychometric approach consisting of directly administered tasks, concurrent observation of the child’s skills and caregiver recall; which offers several advantages over each approach used alone in the characterization of a child’s neurodevelopment skills. Despite its fewer items, shorter administration time, and administration by non-specialists rather than specialists, it has shown good agreement with the BSID III edition (intraclass correlation coefficients between 0.745 and 0.883 [*p*<0.001] for all sub scales) [33] and substantial inter-rater (*k* = 50.70, 95% CI: 0.47–0.88) and test-retest reliability (*k* = 50.79, 95% CI: 0.48–0.96) [33]. It is administered and scored in 15 minutes, and is amenable to administration in the field by trained non-specialists [35]. Its norms are based on international standards of child development, constructed according to the WHO Multicentre Growth Reference Study’s prescriptive approach, rather than on descriptive population-specific references [36].
8. Laboratory assessment: CHIKV infection status was defined by IgG and IgM ELISA results in mothers and children. All maternal and child serum samples collected during the assessment were thawed and initially screened by IgG ELISA (InBios CHIKjj Detect IgG ELISA) to confirm CHIKV infection. All children with positive IgG results were further tested for recent exposure by IgM ELISA (InBios CHIKjj Detect IgM kit) to rule out infection in the previous 6–12 months. As 99.7% of transplacental IgG disappears by two years [20], children who were IgG positive were considered CHIKV infected. Children born to mothers who were pregnant during the CHIKV outbreak in Grenada (July through November 2014), who tested IgG positive, and who reported joint pain in addition to either fever, body pain, or rash were considered CHIKV exposed [6]. The following groups were established for the present study based on CHIKV ELISA test results:
  1. IgG- mothers were considered CHIKV uninfected.
  2. IgG+ mothers were considered CHIKV infected.
  3. IgG- children were considered CHIKV uninfected.
  4. IgG+ children were considered CHIKV infected.
  5. IgG- children born to IgG+ mothers who reported CHIKV symptoms during pregnancy were considered CHIKV exposed-uninfected.
  6. IgG+ children born to IgG+ mothers who reported CHIKV symptoms during pregnancy were considered CHIKV exposed-infected.
  7. IgG- children born to IgG- mothers were considered CHIKV unexposed *in utero*.
  8. IgG+ children born to IgG- mothers and IgG+/IgM+ children were considered CHIKV infected postnatally via mosquito bite.

### Data analysis

The data was analyzed using the Statistical Package for the Social Sciences (SPSS) v. 25 (IBM Corp). INTER-NDA mean domain scores were calculated for cognitive, fine motor, gross motor, language and behaviour (positive and negative) domains using the procedure described by Fernandes, Villar and colleagues [36]. Mean scores were converted to standardized scaled scores and compared with the international INTERGROWTH-21^st^ Project INTER-NDA standards [36]. Kolmogorov-Smirnov tests were used to determine if the distribution of INTER-NDA subscale scores across the groups being compared were equal, and thus, should be analyzed via parametric or non-parametric statistical tests. All of the score distributions were equivalent, so parametric tests were run for all analyses. The tests consisted of general linear models to determine child neurodevelopmental performance differences on the INTER-NDA. The following four analyses were performed:

1. Mothers CHIKV status: IgG+ versus IgG-–General linear model (including symptom duration and number of symptoms experienced by mother)
2. Children CHIKV status: IgG+ versus IgG-–General linear model
3. Children postnatal CHIKV status: IgG+ children born to IgG- mothers (Postnatal mosquito-borne infection) vs. IgG- children–General linear model
4. CHIKV illness during pregnancy broken down by trimester of infection: IgG+ children vs. IgG- children born to IgG+ mothers who report symptoms first, second, or third trimester)–General linear model

Children born to mothers who were infected within 14 days before childbirth or intrapartum (N = 1), defined by symptoms reported in this period, were excluded from the above analyses given the possibility the mother had not developed a strong enough IgG antibody response to be transferred to the offspring, which precluded child classification.

To determine if the mothers’ symptoms of CHIKV were associated with child neurodevelopment, the mothers’ self-reported symptom duration and number of symptoms experienced during pregnancy were examined. Mothers with confirmed IgG+ status who reported symptoms ranging from 0–7 days were classified as “short symptom duration”; whereas mothers who reported 8–42+ days were classified as “long symptom duration.” Categories were determined through the use of a median split. Number of symptoms experienced by IgG+ mothers was also categorized by a median split into: “few” (0–3 symptoms) and “many” (4–15 symptoms). Mother symptom duration and symptom count were used as independent variables to compare child INTER-NDA domain scores. A Bonferroni correction was implemented for all analyses to account for multiple comparisons across the INTER-NDA domain scores.

## Results

### Participant recruitment

**Fig 1:**
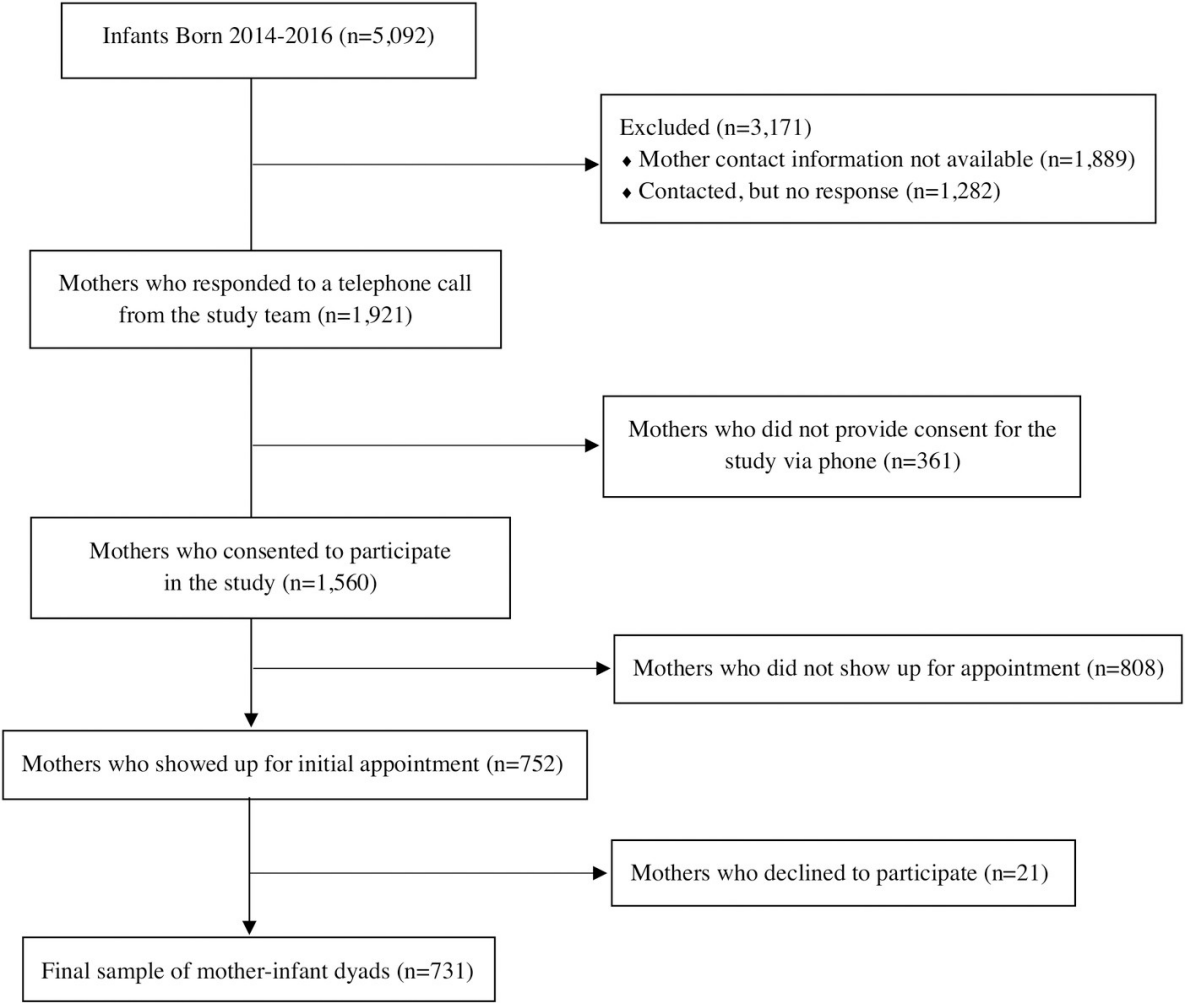
Flow chart of participation recruitment.

**Fig 2:**
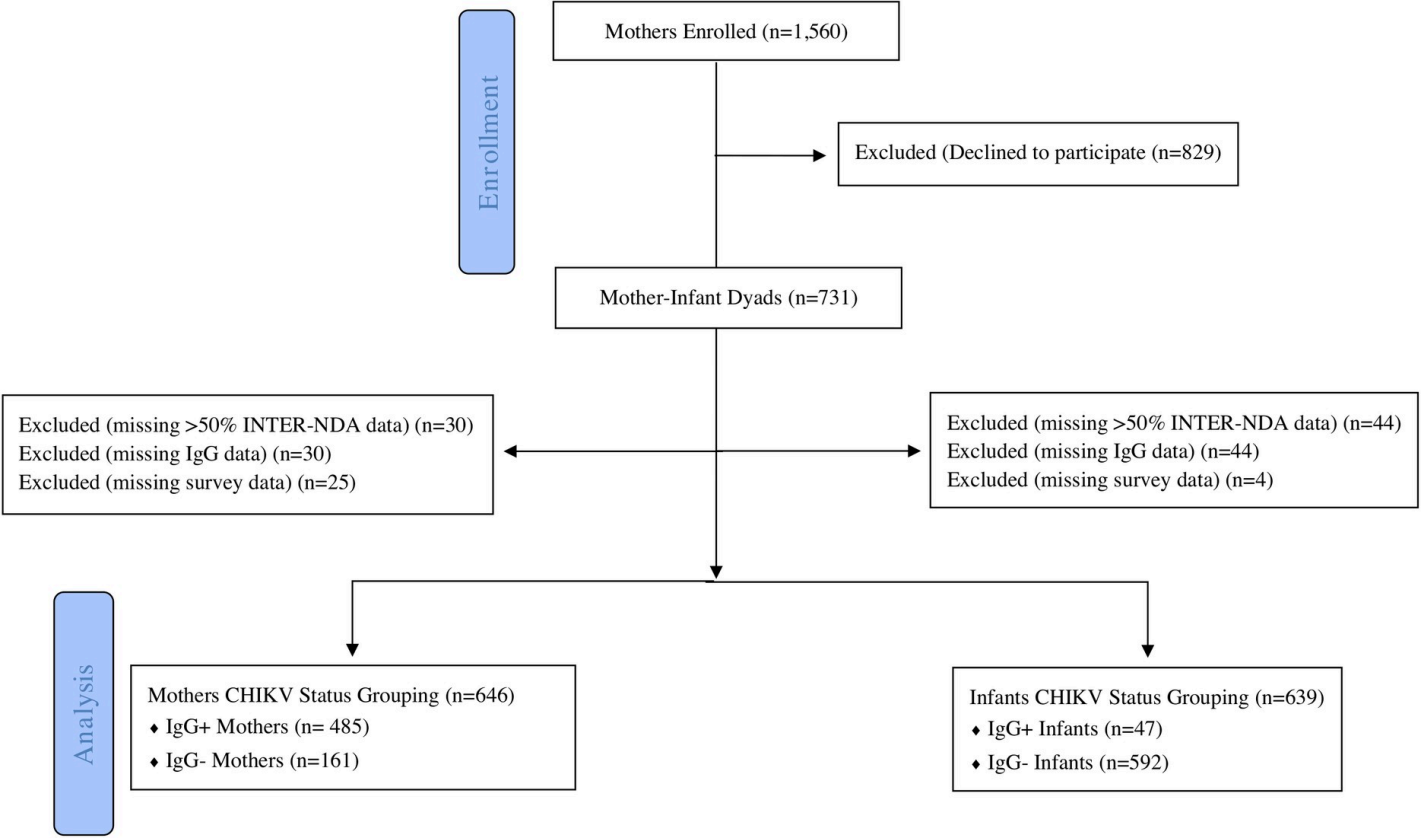
Flow chart of participant breakdown.

The total number of births registered with the Grenada Ministry of Health were 1,782 births in 2014, 1,694 births in 2015, and 1,616 births in 2016. Telephone contacts were obtained from Ministry of Health records for 3,203 of the mothers who gave birth before the outbreak (i.e., January 1 –June 30, 2014), during the outbreak (i.e., July 1 –December 31, 2014) and immediately following the CHIKV outbreak (i.e., January 1 to August 31, 2015). Of those, 1,921 were reached via telephone call, while contact was not made with 1,282 due to outdated phone numbers or no answer. Of those contacted, 1,560 (81.2%) initially agreed to meet with the research team to learn more about the study, but 752 mothers (48.2%) actually attended visits with the research team, and 731 of those (97.2%) consented to participate in the study. After removing participants as a result of missing data (sometimes neuropsychological data was not able to be collected from the children due to distractions or child fatigue), the final cohort for data analysis consisted of 676 mothers (92.3%) and 683 children (93.3%) (Figs 1 and 2).

### Sample characteristics

In total, 731 mother-infant dyads participated in the study. Children with missing responses for more than 50% of the INTER-NDA items were removed from the dataset (n = 30 in the Mothers CHIKV status group and n = 44 in the Infants CHIKV status group). Mothers or children with equivocal or missing IgG results were excluded from the dataset (n = 30 in the Mothers CHIKV status group and n = 44 in the Infants CHIKV status group). Children of mothers with missing survey data were excluded from the dataset (n = 25 in the Mothers CHIKV status group and n = 4 in the Infants CHIKV status group). The samples for analysis consisted of N = 646 in the Mothers CHIKV status group and N = 639 in the Infants CHIKV status group (Fig 2). Results of ELISA testing indicated CHIKV infection of 75.1% (N = 485) of the mothers and 7.4% (N = 47) of the children. Mothers in the study were not tested for IgM, and only children who tested positive for IgG were tested for IgM. None of the children tested positive for CHIKV IgM, indicating prenatal CHIKV infection or remote CHIKV infection in the first 12–18 months of life.

Chi-square and two tailed, independent samples *t*-test analyses indicated that infant gender and age were not significantly different between IgG+ versus IgG- children or IgG+ children born to IgG- mothers versus IgG- children (Table 1). Chi-square and two-tailed, independent samples *t*-test analyses were used to determine the impact of socioeconomic variables (i.e., maternal age, relationship status, education and income) on INTER-NDA scores. Results indicated that mothers of CHIKV exposed-uninfected children were less likely to be in a relationship, X^2^ (1) = 4.76, *p =* .029, and to have lower income, X^2^ (3) = 11.37, *p =* .010 (Table 2). Maternal questionnaire data were also analyzed to determine the presence of covariates that could impact potential differences in INTER-NDA standardized scores between CHIKV-exposed-uninfected versus unexposed and CHIKV infected versus uninfected children. Results indicated that mothers of CHIKV exposed-uninfected children were more likely to report illness/health problems during pregnancy, X^2^ (1) = 9.44, *p =* .002 (Table 3), which was expected given the high symptomatic rate seen with CHIKV infection. These variables were incorporated into the main general linear model results analyses as covariates and did not significantly impact the potential differences in INTER-NDA scores between CHIKV-exposed-uninfected versus unexposed and CHIKV infected versus uninfected children, as reported below.

**Table 1:** Sample characteristics for CHIKV Infected vs. Uninfected Children: Means and SDs.

Infant	IgG+ Child N = 47	IgG- Child N = 592	*p*
Gender			
Female	14 (54%)	206 (52%)	.140
Male	26 (46%)	231 (48%)	
Age (weeks)	107 (16.13)	108 (17.43)	.668
Infant	Child Infected at postpartum (IgG+ child / IgG- mother) N = 19	CHIKV Uninfected Child (IgG-) N = 592	*p*
Gender			
Female	7 (41%)	212 (46%)	.678
Male	10 (59%)	246 (54%)	
Age (weeks)	107 (18.90)	108 (17.30)	.959

**Table 2:** Sample characteristics for CHIKV Exposed-Uninfected vs. Unexposed Children: Means and SDs.

Infant	CHIKV Exposed N = 149	CHIKV Unexposed N = 161	*p*
Gender			
Female	68 (46%)	66 (53%)	.253
Male	79 (54%)	58 (47%)	
Age (weeks)	109 (15.23)	108 (17.86)	.750
Mother	CHIKV Exposed N = 149	CHIKV Unexposed N = 161	*p*
Age (years)	30 (5.43)	29 (5.73)	.554
Relationship Status			
In a relationship	79 (53%)	100 (65%)	.029
Not in a relationship	70 (47%)	53 (35%)	
Education Level			
Primary	9 (8%)	18 (15%)	.514
Secondary	87 (74%)	83 (69%)	
Tertiary	21 (18%)	20 (16%)	
Income			
Under $1000	36 (30%)	19 (17%)	.010
$1000 - $2000	22 (18%)	33 (30%)	
$2001 - $3000	33 (28%)	21 (19%)	
$3000+	28 (24%)	38 (34%)	

**Table 3:** Comparison of pregnancy, maternal health, home, and environmental factors by maternal infection status.

Measure	CHIKV Exposed N = 149	CHIKV Unexposed N = 161	*p*
Baby born: Early Term Late	9 (6%) 122 (82%) 18 (12%)	14 (9%) 125 (82%) 13 (9%)	.387
Illnesses/Health problems during pregnancy Yes No	71 (48%) 76 (52%)	47 (30%) 105 (70%)	.002
Alcohol consumption Yes No	6 (4%) 143 (96%)	14 (9%) 138 (91%)	.071
Illegal drug consumption* Yes No	5 (3%) 143 (97%)	4 (3%) 147 (97%)	.712
Place of birth: Home Clinic Hospital In Transit	2 (1%) 0 (0%) 147 (99%) 0 (0%)	2 (1%) 2 (1%) 148 (96%) 1 (1%)	.399
Mode of delivery: Vaginal Caesarean	138 (93%) 11 (7%)	140 (92%) 13 (8%)	.720
Complications during birth Yes No	17 (11%) 132 (89%)	25 (16%) 128 (84%)	.216
Infant problems two weeks after birth Yes No	35 (24%) 111 (76%)	31(20%) 121 (80%)	.457
Infant problems months after birth Yes No	25 (18%) 116 (82%)	15 (11%) 126 (89%)	.088
Infant feeding: Breast Fed Bottle Fed Both	39 (26%) 7 (5%) 103 (69%)	43 (28%) 9 (6%) 102 (66%)	.832
Postpartum depression experienced: Yes No	28 (19%) 120 (81%)	18 (12%) 135 (88%)	.085
Food Security in the home: Food Secure Food Insecure (Moderate) Food Insecure (Severe)	92 (62%) 29 (19%) 28 (19%)	107 (66%) 32 (20%) 22 (14%)	.464
	Mean (SD)	Mean (SD)	*p*
Weeks gestation at birth	39.38 (1.84)	39.16 (2.09)	.386
Months of breastfeeding (1–12 Months)	9.06 (3.74)	9.27 (3.55)	.622
Maternal General Health**	17.75 (3.18)	17.88 (3.94)	.754
Maternal Social Support***	11.95 (6.13)	12.75 (5.09)	.222
HOME Record***	34.48 (6.26)	34.09 (7.19)	.617

* Marijuana was the only illegal drug reported. No use of steroids, cocaine/crack or heroine.

** Higher General Health score indicates worse general health.

*** Higher Social Support score indicates more supportive environment, higher HOME score indicates a more balanced home environment.

### CHIKV Exposed-uninfected and unexposed Children

**Fig 3:**
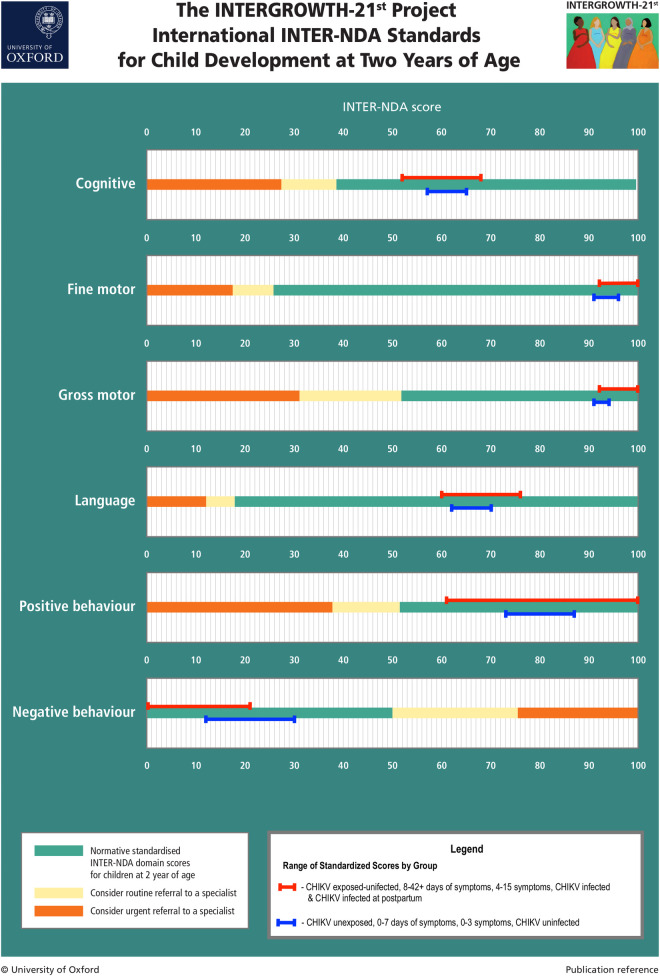
INTERNDA Child Development Standards with group domain scores: Blue lines represent the range of mean standard scores across CHIKV unexposed/uninfected children and children born to mothers with fewer symptoms and shorter symptom duration. Red lines represent the range of mean standard scores across CHIIKV exposed/infected children and children born to mothers with greater symptoms and longer symptom duration. All mean standardized scores from the current study fall within the normative range across all INTER-NDA domain scores. Note: For all INTER-NDA domains, higher scores indicate better outcomes except for negative behaviour where lower scores indicate better outcomes. Figure adapted with permission from Fernandes, Villar, and colleagues (36).

There were no significant differences between CHIKV exposed-uninfected and unexposed children across INTER-NDA cognition, fine motor, gross motor, language, and positive behaviour standardized scores. A significant difference emerged in negative behaviour standardized scores between CHIKV exposed-uninfected versus unexposed children, F(1) = 25.59, *p* < .001 (Table 4). However, all standardized scores for both groups, including for negative behavior, were within the normative range according to the international INTERGROWTH-21^st^ Project INTER-NDA standards [36], suggesting no neurodevelopmental delay in both groups across all domains (see Fig 3).

**Table 4:** Mean differences on standardized INTER-NDA domain scores between CHIKV exposed-uninfected and CHIKV unexposed children at two years of age.

INTER-NDA Domains	CHIKV Exposed Children (N = 149)	CHIKV Unexposed Children (N = 161)	
	INTERNDA Score /100 [CI]	INTERNDA Score /100 [CI]	
Cognition	61.64 [59.09–64.19]	62.57 [60.30–64.87]	*p* = .588
Fine Motor	94.25 [92.32–96.20]	94.90 [93.03–96.77]	*p* = .614
Gross Motor	92.69 [90.66–94.71]	91.73 [89.77–93.67]	*p* = .463
Language	65.68 [62.62–68.73]	63.37 [60.17–66.53]	*p* = .325
Positive Behaviour	83.29 [79.95–86.62]	80.90 [77.60–84.20]	*p* = .346
Negative Behaviour	11.91 [9.35–14.47]	27.00 [21.40–32.65]	*p* = .000

Neither CHIKV symptom duration nor number of CHIKV symptoms in mothers were significantly associated with children’s INTER-NDA standardized domains scores (Tables 5 & 6).

**Table 5:** Mean differences on standardized INTER-NDA domain scores between children born to mothers with short (0–7 days) and long (8–42+ days) symptom duration during pregnancy.

INTER-NDA Domains	Symptom duration: 0–7 Days (N = 62)	Symptom duration: 8–42+ days (N = 94)	
	INTERNDA Standardized Domain Score Mean [CI]	INTERNDA Standardized Domain Score Mean [CI]	
Cognition	59.70 [55.70–63.70]	63.20 [60.27–66.10]	*p* = .153
Fine Motor	92.67 [89.53–96.37]	94.80 [92.40–97.20]	*p* = .368
Gross Motor	91.57 [88.03–95.10]	92.23 [89.50–95.00]	*p* = .761
Language	66.00 [61.33–70.70]	68.17 [64.80–71.57]	*p* = .445
Positive Behaviour	79.65 [73.25–86.05]	85.75 [82.05–89.45]	*p* = .080
Negative Behaviour	12.05 [7.95–16.20]	14.05 [9.45–18.70]	*p* = .546

**Table 6:** Mean differences on standardized INTER-NDA domain scores between children born to mothers with fewer (i.e., 0–3) versus more (i.e., 4–15) symptoms during pregnancy.

INTER-NDA Domains	0–3 Symptoms (N = 73)	4–15 Symptoms (N = 83)	
	INTERNDA Standardized Domain Score Mean [CI]	INTERNDA Standardized Domain Score Mean [CI]	
Cognition	61.07 [57.93–64.23]	62.43 [58.90–65.97]	*p* = .572
Fine Motor	96.03 [93.77–98.30]	92.33 [89.23–95.43]	*p* = .062
Gross Motor	90.77 [87.53–94.03]	93.03 [90.13–95.93]	*p* = .305
Language	65.00 [61.00–69.03]	69.33 [65.60–73.10]	*p* = .118
Positive Behaviour	82.60 [77.65–87.55]	83.95 [79.25–88.65]	*p* = .696
Negative Behaviour	16.05 [10.90–21.25]	10.80 [6.90–14.75]	*p* = .105

### CHIKV Infected and Uninfected Children

No significant differences were observed on INTER-NDA standardized domain scores between CHIKV infected (IgG+) and CHIKV uninfected (IgG-) children (Table 7).

**Table 7:** Mean differences on standardized INTER-NDA domain scores between CHIKV infected vs. CHIKV uninfected children.

INTER-NDA Domains	CHIKV Infected Children (N = 47)	CHIKV Uninfected Children (N = 592)	
	INTERNDA Standardized Domain Score Mean [CI]	INTERNDA Standardized Domain Score Mean [CI]	
Cognition	63.20 [59.27–67.13]	61.80 [60.57–63.03]	*p* = .542
Fine Motor	96.27 [93.80–98.73]	94.00 [92.97–95.03]	*p* = .228
Gross Motor	94.53 [91.10–98.00]	91.13 [90.00–92.23]	*p* = .097
Language	66.77 [61.20–72.33]	62.77 [61.10–64.40]	*p* = .192
Positive Behaviour	80.00 [73.15–86.60]	78.90 [77.00–80.80]	*p* = .760
Negative Behaviour	20.20 [11.45–28.95]	30.05 [27.15–32.90]	*p* = .064

### Postpartum CHIKV Child Infection

IgG+ children born to IgG- mothers and IgG+/IgM+ children were categorized as postnatal (i.e., mosquito-borne) CHIKV infection. These children were compared to CHIKV uninfected children across the INTER-NDA subdomains, and no significant differences were detected (Table 8).

**Table 8:** Mean differences on standardized INTER-NDA domain scores between children infected with CHIKV postpartum vs. CHIKV uninfected children.

INTER-NDA Domains	Child Infected at Postpartum (N = 19)	CHIKV Uninfected Child (N = 592)	
	INTERNDA Standardized Domain Score Mean [CI]	INTERNDA Standardized Domain Score Mean [CI]	
Cognition	66.17 [60.47–71.83]	61.80 [60.57–62.97]	*p* = .214
Fine Motor	96.50 [92.87–100.00]	94.10 [93.10–95.07]	*p* = .406
Gross Motor	93.57 [87.30–99.83]	91.30 [90.20–92.37]	*p* = .475
Language	64.93 [55.00–74.83]	63.33 [61.80–64.57]	*p* = .678
Positive Behaviour	80.00 [72.10–87.85]	78.95 [77.00–80.75]	*p* = .840
Negative Behaviour	14.50 [2.20–26.70]	29.90 [27.05–32.70]	*p* = .060

### Trimester of CHIKV Infection

CHIKV IgG+ mothers who reported CHIKV symptoms in their 1^st^, 2^nd^, and 3^rd^ trimester were selected to determine if the trimester of CHIKV infection had a significant effect on child neurodevelopment at two years of age. No significant differences were found on INTER-NDA standardized scores regardless of trimester of maternal CHIKV infection (Table 9).

**Table 9:** Mean differences on normative standardized INTER-NDA domain scores between children born to mothers CHIKV infected in first, second, and third trimester.

INTER-NDA Domains		CHIKV Infected Children	CHIKV Uninfected Children	
		INTERNDA Standardized Domain Score Mean [CI]	INTERNDA Standardized Domain Score Mean [CI]	
Mother first Trimester Reported Infection	Cognition	64.10 [64.10–64.10]	64.63 [60.80–68.50]	*p* = .954
	Fine Motor	100.00 [100.00–100.00]	95.33 [92.53–98.10]	*p* = .508
	Gross Motor	100.00 [100.00–100.00]	93.33 [90.33–96.30]	*p* = .379
	Language	76.37 [54.80–97.97]	70.43 [65.73–75.10]	*p* = 617
	Positive Behaviour	100.00 [100.00–100.00]	86.60 [81.40–91.75]	*p* = .309
	Negative Behaviour	0.00 [00.00–00.00]	14.50 [7.90–21.05]	*p* = .385
Mother Second Trimester Reported Infection	Cognition	63.47 [53.83–73.13]	61.40 [57.00–65.80]	*p* = .712
	Fine Motor	100.00 [100.00–100.00]	93.67 [89.63–97.73]	*p* = .215
	Gross Motor	92.57 [80.53–100.00]	91.13 [85.43–96.80]	*p* = .842
	Language	60.33 [37.10–83.60]	64.60 [58.83–70.37]	*p* = .593
	Positive Behaviour	90.00 [76.70–100.00]	87.35 [82.70–92.00]	*p* = .666
	Negative Behaviour	16.65 [2.45–38.06]	14.15 [7.20–21.20]	*p* = .788
Mother Third Trimester Reported Infection	Cognition	53.83 [41.20–66.47]	57.93 [50.87–64.97]	*p* = .640
	Fine Motor	92.57 [78.47–100.00]	91.73 [86.47–97.00]	*p* = .900
	Gross Motor	96.27 [86.77–100.00]	91.73 [87.87–95.60]	*p* = .354
	Language	71.27 [61.93–80.63]	66.37 [60.10–72.63]	*p* = .524
	Positive Behaviour	61.65 [33.95–89.35]	73.10 [62.90–83.35]	*p* = .382
	Negative Behaviour	7.05 [5.00–46.60]	11.40 [7.05–15.75]	*p* = .156

## Discussion

In this study, we observed no meaningful difference in neurodevelopment at two years of age between children exposed to but uninfected by CHIKV *in utero* and unexposed children. We also did not find a difference in neurodevelopment between those infected and those not infected with CHIKV *in utero*. Timing of maternal infection with CHIKV during gestation and the severity of maternal chikungunya disease were not associated with neurodevelopmental outcomes. Reassuringly, all children plotted within the normative ranges for the international INTERGROWTH-21^st^ Project INTER-NDA standards, confirming that all children exposed and not exposed to CHIKV during the prenatal and postnatal periods were developmentally healthy at two years of age. These results are consistent with previous findings in La Réunion, which only showed neurodevelopmental impacts in children who were infected with CHIKV during the intrapartum period [16,19]. In this study we were unable to confirm this finding because only one mother was infected with CHIKV during the intrapartum period. Our results indicate that CHIKV, a high-titer virus that often causes severe symptoms and a high symptomatology rate of 75%, does not impact the fetus during development in the first two years of life. This is an important finding for public health officials and women of reproductive age in CHIKV-endemic regions since CHIKV can be transmitted in a human-mosquito-human cycle resulting in widespread transmission in densely populated areas. CHIKV may be unable to cross the placental barrier during pregnancy, leading to very low infection rates during gestation [37]. There is no reliable epidemiological data that links infection in the first trimester of gestation with an increased risk of miscarriage or congenital malformation. In the second trimester, while it is unlikely for CHIKV to cross the placental barrier, there have been very few reported cases of fetal deaths during periods of deep trophoblast invasion, where accidental infections may be possible. In the third trimester, although there were cases of stillborn fetuses associated with chikungunya fever, none tested positive for the virus. These results support the rarity of gestational infection [16].

Results from the current study also demonstrated that children infected with CHIKV postpartum via *Ae*. *aegypti* vectors during the first two years of life, as determined by CHIKV ELISA tests (i.e., IgG+ children born to IgG- mothers) do not show different neurodevelopmental outcomes at two years of age compared to children who were not infected with CHIKV. While neonates may experience physiological symptoms associated with postnatal CHIKV infection (e.g., febrile seizures, diarrhea, vomiting, arthralgia/arthritis, acrocyanosis, uremia, lymphopenia, cytolysis, etc.) [10, 13, 15–17, 19, 21, 24, 25], existing evidence indicates that the vast majority of older children will recover and not experience any long-term impact of CHIKV infection [38–44]. Results from the current study indicate no negative impact of postnatal CHIKV infection on neurodevelopment in children at two years of age. This finding applies specifically to children infected with CHIKV via mosquito bite within the first 12–18 months after birth, since none of the children in the present study tested positive for recent CHIKV infection (i.e., the last 6–12 months) via IgM ELISA. It should be noted that the sample of children infected with CHIKV in the postpartum period, as confirmed by IgG+ ELISA, was small and thus, further studies should be carried out to confirm the lack of neurodevelopmental impact at two-years of age (and older) in postnatally infected children.

The lack of differences in neurodevelopment at two years of age between children exposed to but uninfected with CHIKV versus children unexposed to CHIKV and children infected versus uninfected with CHIKV is encouraging, but we acknowledge the limitation that we were unable to assess the impact of intrapartum CHIKV infection on child neurodevelopment given that only one woman reported intrapartum infection with CHIKV in the study. In addition, the precise timing and mechanism of infection could not be determined in the children in this study as even those who were IgG+ born to IgG+ mothers (i.e., exposed-infected) could have been infected with CHIKV in the first two years of life (i.e., postpartum rather than prenatal) and symptoms of illness may not have been identified as CHIKV infection at the time. There is an inverse correlation of timing of infection in pregnant women and persistence of transplacental IgG in the infant’s blood (earlier in pregnancy the infection, the longer the persistence of transplacental IgG) [45]. The proportions of first trimester and second trimester infections on Réunion and Grenada are very close (74% and 70%), enabling extrapolation of the Réunion finding that approximately 5% of infants whose mothers were infected during the first or second trimester of pregnancy still had maternal transplacental IgG at 22 months [45]. This previous data, combined with a single case of peripartum infection reported in the present study, combined with evidence that CHIKV very rarely crosses the placental barrier, leads us to estimate that approximately 95% (n = 27) of IgG+ children born to IgG+ mothers in the current study were infected postpartum via mosquito bite. Approximately 5% (n = 1) of the IgG+ infants born to IgG+ mothers could be classified as exposed-uninfected with long-lasting persistence of maternal transplacental IgG.

While no clinically significant impact on neurodevelopment was demonstrated among children exposed or infected with CHIKV, this assessment was conducted at age two, which may or may not be the optimal age for determining neurodevelopmental differences, either due to compensation before age two, or to more subtle differences as children age. Further studies will need to follow CHIKV-exposed and infected children to answer these questions.

An additional limitation in comparing the results of the present study against those from the CHIKV outbreak in La Réunion is the use of different neurodevelopment assessment tools: The INTER-NDA in the current study and the Scale of Psychomotor Development in Children, also known as the Brunet-Lezine, in the studies from La Réunion [14]. However, unpublished data from the present study demonstrated strong agreement in normal range scores between the INTERNDA and Brunet-Lezine, indicating that both scales assess child development similarly at two years of age. The use of unique assessment tools is unlikely to play a key role in the different results reported in the Réunion studies and the present study.

Finally, the study was subject to some limitations with laboratory assays. Detection of antibodies by serological assay fails to identify acute infections and is only indicative of exposure retrospectively. IgM is often used as a proxy for the detection of acute or recent infections without the detection of viral genomic material by polymerase chain reaction (PCR), yet the duration of IgM expression and persistent circulation varies by individual. The detection of IgG and IgM does not indicate the experience of disease; thus, questionnaire data may not represent the presence of symptoms for each seropositive participant. Additionally, although serological assays are subject to cross-reactivity, a large-scale, island-wide CHIKV outbreak was documented in Grenada, and it was unlikely that other alphaviruses were responsible for the antibody response in our participants. All kits used in this study were purchased from InBios directly and were validated by InBios upon production.

Despite the potential limitations, this study represents the first effort to confirm the impact of CHIKV exposure and/or infection on neurodevelopment in children born during or shortly after an outbreak of the virus in an immunologically naïve population, and advances knowledge about the lack of significant impact of exposure and/or post-natal infection of CHIKV on neurodevelopment in two-year-old children. The results of the study should provide reassurance for parents and early child development specialists in tropical regions endemic to the *Aedes spp*. mosquito, who may be concerned about the potential impact of CHIKV infection in the age of Zika virus infection and its potentially severe neurodevelopmental outcomes in children [46,47].

### Conclusion

This study assessed two-year old children born to mothers who were infected and not infected with CHIKV during the 2014 outbreak in Grenada to determine the neurodevelopmental impact of CHIKV infection. We observed no significant impact of *ante* or postpartum CHIKV exposure on child neurodevelopment at two years of age as measured by the INTER-NDA. We also observed no impact of postnatal CHIKV infection on child neurodevelopment at two years of age. Neither maternal symptom duration nor the number of symptoms experienced by mothers impacted child neurodevelopment at two years. These results confirm that postnatal exposure and/or infection with CHIKV does not adversely impact neurodevelopment in children up to two years of age.
